# The many “costs” of transportation: Examining what cancer caregivers experience as transportation obstacles

**DOI:** 10.1002/cam4.6351

**Published:** 2023-07-23

**Authors:** Maria D. Thomson, Courtney Harold Van Houtven, Rebecca Xu, Laura A. Siminoff

**Affiliations:** ^1^ Department of Health Behavior and Policy Virginia Commonwealth University Richmond Virginia USA; ^2^ Department of Population Health Sciences Duke University Durham North Carolina USA; ^3^ Durham ADAPT, Durham Veterans Affairs Medical Center Durham North Carolina USA; ^4^ Virginia Commonwealth University School of Medicine Richmond Virginia USA; ^5^ Department of Social and Behavioral Sciences Temple University Philadelphia Pennsylvania USA

**Keywords:** behavioral science, cancer management, psychosocial studies, quality of life

## Abstract

**Background:**

Transportation has been identified as a specific source of burden for cancer caregivers. This study examined cancer caregivers' subjective experiences and objectives costs associated with transportation over a 6‐month period of providing end‐of‐life care to a family member or friend.

**Methods:**

This was a multi‐site longitudinal, prospective cohort study that followed 223 caregiver–patient dyads. Data were collected using biweekly, semi‐structured interviews for up to 6 months and collection of all caregiving related receipts. Interviews were coded and analyzed using a comparative, iterative analysis and actual out of pockets costs were described using descriptive statistics.

**Results:**

Over the 6‐month study period most caregivers (*n* = 143; 74%) discussed transportation at one or more timepoints. Average biweekly transportations costs to caregivers were $43.6. Caregivers described (*n* = 56; 39%) multiple direct and indirect costs of transportation, and 58% (*n* = 84) discussed the need for transportations services or assistance at the institutional level.

**Conclusions:**

Caregivers described the multifaceted costs of transportation they experienced which are in line with previous work. Alongside descriptions of direct costs, caregivers described key opportunity costs, such as personal and work time forgone to transporting patients. Caregivers also made suggestions for institutional and/or civic based solutions to facilitate reliable modes of transportation, rather than individual‐level intervention.

## 1 INTRODUCTION

Access to quality cancer care can be limited by travel burden. Every year in the United States, approximately 3.6 million people forgo nonemergent medical care because of transportation obstacles including distance to healthcare facilities; vehicle operation, maintenance and parking costs, and poor availability and reliability of public transportation.^1^ For cancer patients this is a critical problem as they often require frequent visits for treatment and follow‐up. Delayed treatments, missed appointments, and discontinuous cancer care can exacerbate cancer management and compromise health outcomes, as therapy is typically time intensive and requires multiple visits over a period of weeks or months but missed appointments and nonadherence to the schedule has been shown to decrease its effectiveness.^1^, ^2^, ^3^

Distance from treatment facilities has been associated with increased cancer diagnostic, treatment delay and lower participation in clinical trials.^4^, ^5^ In a 7‐year cohort, patients with any type of suspected cancer who did not attend their initial referral appointments had an early mortality risk compared to those who did attend (31.3% vs. 19.2%) and distance to the medical center was a significant predictor of nonattendance.^6^ The problem of transportation has long been recognized by national entities dedicated to improving patient care. In 1981, the American Cancer Society (ACS) implemented the Road to Recovery, a transportation service for cancer‐related appointments via a volunteer network in all 50 states. Since its implementation, it has served over 400,000 patients, with an additional 7500 patients in rural areas assisted via transportation grants.^7^ While beneficial, these programs have important disadvantages. Notably, drivers are untrained in medical transport and require patients to be able to get in and out of the vehicle themselves.^8^ Lyft has additionally partnered with National MedTrans, a nonemergency medical transportation benefit management company to arrange rides for Medicaid and Medicare Advantage participants.^9^ Data have shown that the implementation of this program has decreased average wait times and average per‐ride costs^9^ but it is unclear if it successfully reduces missed appointments.^10^ While showing value, these programs are not universal and reach only a small portion of the population incurring transportation costs for cancer care. In the absence of these options, family caregivers are often responsible for assisting patients to get to and from their medical visits.

Transportation adds to the financial burden of cancer and contributes to the indirect costs associated with loss of time and productivity for those receiving cancer treatment.^11^ The average out‐of‐pocket costs for transportation to radiotherapy appointments among breast cancer patients living at home was $323 for a period of 4–5 weeks.^12^ The indirect costs of lost revenue and productivity was estimated to be $344 million in a study of employed breast cancer survivors aged 18–34.^13^ Cancer patients face multifaceted challenges to care that require reliable methods of transportation, including strict chemotherapy regimens, frequent visits to the emergency department in the early stages of diagnosis, and access to support groups for emotional well‐being.^14^, ^15^, ^16^ Thus, they often require assistance from family and friend caregivers to accompany them to appointments and provide transportation. There is extensive literature that details the obstacles faced by cancer patients in accessing care yet understanding the burden that this places on cancer caregivers is less understood. Caregivers for patients receiving end‐of‐life care will desire spending more personal time with their loved ones, rather than time spent navigating the transportation system and managing financial costs of transport. From the caregiver perspective, transportation costs may pose an additional stressor in their lives, affecting their emotional and mental health. In this study, we examine cancer caregivers' subjective experiences and objectives costs associated with transportation as a specific type of cancer caregiver burden.

## 2 METHODS

This was a multi‐site longitudinal, prospective cohort study that followed 223 caregiver‐patient dyads. Dyads were interviewed biweekly for up to 6 months or until 1 month after patient death. Data were collected biweekly and in‐person from caregivers in their homes using semi‐structured, in‐depth qualitative interviews, quantitative surveys, monthly structured observation, and weekly diary completion. Patients completed a brief survey. All dyads participated in a baseline (T1) interview, an initial observation and up to 11 subsequent waves of data collection. Dyads participated in an average of 5 (range 1–11) interviews. This study was approved by the university institution review board #HM20003319. Participants provided written informed consent prior to participation. The primary aims of the study were to identify and describe caregivers' lived experiences of subjective and objective burden. While caregivers were queried about many different tasks, transportation emerged as a salient for the majority of caregivers in both subjective reports of lived experiences and objective reports of out of pocket (OOP) costs, therefore the present work focuses on this specific issue and how it was experienced by caregivers providing care at end‐of‐life.

### 2.1 Participants

Patients and their caregivers were recruited from two NCI‐designated Cancer Centers in Virginia and Pennsylvania as part of a longitudinal study. Patients were older than 25 years and diagnosed with an advanced (Stage IV) solid tumor cancer with a life expectancy of less than 12 months, as assessed by their oncologist and could identify a primary caregiver who was willing to participate. Patients who lived in residential facilities, such as a nursing home, were excluded. Informal caregivers were identified by patients. The inclusion criteria for the caregivers were as follows: (1) over 18 years old, (2) able to speak and read English, and (3) identified as the primary, unpaid caregiver. Participants were compensated for their time. Study procedures were approved by relevant institutional review boards.

### 2.2 Recruitment

Preliminary eligibility was determined using patient electronic medical records and case conferences from three oncology clinics in Virginia and Pennsylvania. A HIPAA waiver of authorization was obtained for identification purposes. Patient oncologists were contacted to confirm eligibility and to request permission to contact the patient. Brief screening questionnaires delivered via telephone were used to determine interest and presence of a caregiver. Although recruitment began with patients and a patient‐caregiver dyad was enrolled, caregivers were the primary focus for this study.

### 2.3 Interviews

Semi‐structured interviews were conducted biweekly and in person at the participants' home, lasted between 45 and 90 min and were conducted by trained research staff. The interviews were designed to obtain longitudinal descriptions of caregivers' experiences of the caregiving tasks they perform and included questions about subjective caregiver burden and the type and frequency of caregiving tasks. Interviews were transcribed verbatim and coded. The current analysis is limited to qualitative descriptions of caregiver's experiences with transportation over the course of providing care and the OOP costs collected through receipts. Caregivers were asked to discuss all tasks performed and the level of burden experienced (see Supplemental Material) but those are not reported here.

### 2.4 Measures

Caregiver demographics included age, gender, highest level of attained education, and annual household income. The relationship between the caregiver and patient was recorded.

OOP Expenses: Caregivers and patients were provided a box and were asked to keep all receipts and bills related to the patient's care over the project period. These included the patient's healthcare utilization (e.g., co‐pays, treatment costs, transportation), caregiver's own healthcare utilization related to caregiving (e.g., a primary care visit to address strain or injury), and supplementary services that assist the caregiver (e.g., house cleaning, massage therapy, childcare). At each biweekly meeting, the receipts and bills were recorded by a study team member in a spreadsheet noting date, reason for expense (e.g., prescriptions, equipment), payer (caregiver or patient) and payee. Caregivers were asked if there were any additional bills/receipts not present in the box. Mileage was calculated using the IRS‐determined rate at the time of collection ($0.535–$0.58/mile). Expenses were checked and adjudicated by the research team to ensure that they could be reasonably attributed to the caregiving role. Expenses were categorized into broad types of costs based and summed across all time points. OOP costs collection began at the initial study visit and were recorded at study visits 2–12.

#### 2.4.1 Analysis

Interview data were coded using an iterative thematic approach.^17^, ^18^, ^19^ A coding guide previously used in cancer caregiving studies with lung and lymphoma caregivers was adapted for use in the current study. Ten caregiver interviews were iteratively coded by the coding team (Co‐PI and master's level research assistants). Fit of the original codes was examined; any modifications or new codes were added. This process resulted in a revised codebook that was tested on a new set of 10 caregiver interviews. Inter‐rater reliability was assessed and found to be adequate. The remainder of the interviews were coded with 10% double coded to monitor coder drift. Weekly coding meetings were used to identify discrepancies which were discussed until consensus was reached.

To describe transportation costs, we present the descriptive characteristics of caregivers who reported any transportation costs, and compared caregivers who spent more than the median biweekly transportation cost to those caregivers who spent less than the median biweekly transportation costs. We perform two sample *t*‐tests for continuous variables and Fisher's exact test for categorical variables to examine whether descriptive characteristics differ by being a high spender on transportation (above median) or low spender (below median) and evaluate these at the 95% confidence level. Additionally, we summed all transportation costs by study visit, presenting both total transportation costs per study visit, mean transportation costs per study visit and the number of caregivers represented in each study visit graphically. Analyses completed using STATA.^20^

## 3 RESULTS

Of the 875 patients contacted, approximately 23.9% declined participation, 50.3% were ineligible, and 25.4% provided informed consent. Table 1 displays caregiver characteristics divided by those who reported paying above and below the median OOP transportation costs. Caregivers were primarily female (73%), White (56%), and PTs were male (50.9%), White (50.9%); 42.4% (*n* = 97) patients died during the study. Less than 10% (*n* = 11) reported living in a rural area (RUCC 7–9). The most common diagnosis among patients among patients that they were providing care for was lung cancer (25.7%). Over the 24‐week study period most caregivers (*n* = 143; 64%) discussed and *n* = 165 (74%) reported OOP costs for transportation at one or more timepoints. Significantly greater proportions of participants who were White and married reported paying transportation costs greater than the median OOP costs.

**TABLE 1:** Caregiver characteristics comparing caregivers who spent more than the median biweekly transportation cost to those caregivers who spent less than the median.

	Total	Lower median	Upper Median	*p*‐value
	*N* = 165	*N* = 83	*N* = 82	
Caregiver age	55.74 (13.6)	54.79 (14.6)	56.70 (12.5)	0.37
Caregiver gender				1
Male	27%	28%	27%	
Female	73%	72%	73%	
Caregiver race				0.02
White or Caucasian	56%	48%	65%	
Black or African American	39%	49%	29%	
Asian	1%	0%	1%	
Multi‐racial	2%	2%	2%	
Other	1%	0%	2%	
Caregiver Hispanic				0.37
Not Hispanic/Latino	98%	99%	96%	
Hispanic/Latino	2%	1%	4%	
Caregiver marital status				0.002
Married/cohabitating	61%	48%	74%	
Divorced/separated	16%	18%	13%	
Never married	16%	25%	7%	
Widow/widower	7%	8%	5%	
Caregiver ESAS score	33.07 (17.7)	33.53 (18.6)	32.61 (16.8)	0.74
Out of pocket transportation costs (USD)	43.60 (65.73)	9.96 (7.24)	77.65 (79.76)	0

*Note*: Data are presented as mean (SD) for continuous measures and % for categorical measures.

### 3.1 OOP costs for transportation

**FIGURE 1:**
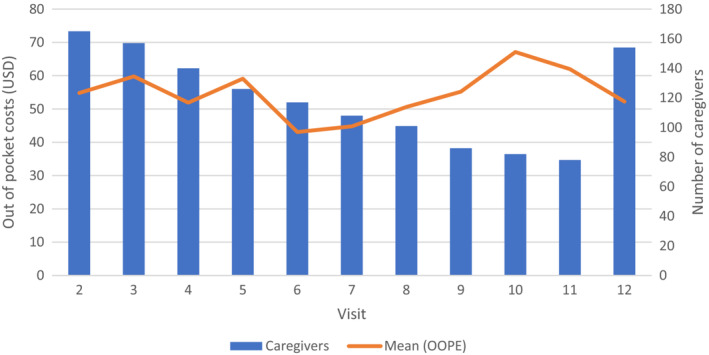
Mean out of pocket transportation costs and number of caregivers by bi‐weekly period.

Of the 165 caregivers reporting OOP transportation costs there were 832 clinic visits recorded during the study period. Over 24 weeks caregivers reported an average of 10 clinic visits with associated transportations expenses. Transportation costs (e.g., mileage, associated receipts) were calculated at each biweekly visit with average reported expenditures of $43.6 (SD 65.73) biweekly, ranging from $0.26 to $457. Figure 1 displays the mean OOP transportation costs calculated per caregiver at each biweekly reporting timepoint. The number of caregivers reporting transportation costs and the total spending decreased over the 24‐week reporting period. However, when viewed as the mean amount spent per caregiver, the average fell each visit with a large increase in the average spent on transportation at final visit at the end of the study period (Figure 1). This increase at the end was likely due to a longer recall period for the closeout visit (timepoint 12; mean = 23.5 days (15.9 SD)) compared to the 2‐week recall period of expenses for study timepoints 2–11. The time elapsed between study timepoint 11 and 12 was longer than for the biweekly visits of the rest of the study.

### 3.2 Caregivers' descriptions of transportation obstacles

We identified four overarching themes in the caregiver descriptions of transportation obstacles. First, caregivers defined transportation costs broadly differentiating between actual dollars spent and opportunity costs involving time and effort removed from other tasks and employment. Second, caregivers described the ease and difficulties experienced trying to find or accept transportation assistance from friends and family. Third, caregivers identified the critical need for institutions and civic entities to be better leveraged to align transportation solutions with family needs, and finally, while caregivers primarily discussed transportation difficulties some reframed these tasks as expressions of care through acts of service. These themes are described below and exemplary quotes for each are provided in Table 2.

**TABLE 2:** Exemplar Quotes by Theme.

The many ‘Costs’ of Transportation (*n* = 56; 39%)
“I mean just driving out there and back and out there and back and out there and back and in the meantime trying to keep my own business going.” ID 2085 (female; sister in law) “Well the driving part would be time, I could do things in the house, clothes washing and I could be preparing meals during that time driving to [city] and sitting there for a number of hours.” ID 1025 (male; partner) “It makes me nervous going into [city], it's like me going by myself and I'm not… I know you're from [city] so it doesn't sound like a major thing but it is when you're on the outskirts and you're not used to big city.” ID 1033(female; sister)
Letting Friends and Family Assist (*n* = 89; 62%).
“My brother's wife is volunteering for this Thursday to go with me, so she actually called and volunteered for it, so that was nice. She was going to go one of the other days that my husband went but I told her that I had it covered but that was nice that she volunteered.” ID 1033 (female; sister) “And appointments, even, like he's got a gazillion friends, but it was always me. Every day, it was always me. Even if it meant leaving my father there to come pick up my son to go back to my father 45 minutes away, when he's got all these friends who love him so much. But he didn't wanna burden them. He was ok burdening me” ID 1003 (female; daughter)
Institutional and/or Civic Solutions to Transportation Support (*n* = 84; 58%).
“Transport her to and from the cancer center would be awesome, or if they could do some of this at the satellites or something that's closer, forty minutes away.” ID 1048 (female; daughter) “What really would be a help is like a c‐van to get her to appointments and stuff. It's basically because if I am not able to take her which I am, my grandmother car is messed up she'll try to get on the bus. And it's like you can't it's too hot now and you're not drinking water and you're very dehydrated if you pass out somewhere we can't find.” ID 2033 (female, daughter)
Expressions of Care Through Acts of Service (*n* = 68F; 47.5%)
“Make sure he gets to the doctors', if I know he has to go see a doctor, I'm gonna make sure he goes see them.” ID 2059 (female; partner) “The only thing that she can't do now is drive and I drive her wherever she wants to go”. ID 1064 (male; partner) “And I think she has appreciated my being available to drive her, especially when she was going through [hospital in different state]. That's a three‐hour drive. She certainly could not have driven herself. So, to be available to take her to run errands.” ID 2035 (female; friend)

#### 3.2.1 The many “Costs” of transportation (*n* = 56; 39%)

Caregivers discussed many problems related to transportation that over time were “costly” to their expenses, time, employment, and/or psychological health. The monetary costs of transportation that were discussed included expensive car maintenance, gas, road tolls. Some caregivers paid others to provide transportation either through use of public transportation, paid drivers (taxi/Uber) or ambulance services.

Outside of monetary expenses, the opportunity costs involved in caregiving were defined as the time commitments required to travel to and from each appointment combined with the difficulties of continuously negotiating a variety of clinic, family and work schedules was also discussed. These opportunity costs were seen by many to take time away from the multitude of other tasks caregivers needed to accomplish including both caregiving tasks, home maintenance and maintaining their own employment.

For many, it was difficult to drive into the cancer center for appointments and treatments. Many caregivers expressed worry about highway driving, unpredictable weather, and concern about possibly driving at night if the appointment wait time was long.

#### 3.2.2 Letting friends and family assist (*n* = 89; 62%)

Many caregivers described transportation‐related support that they received from family and friends. Friends and family helped caregivers by assisting with trips to appointments to provide caregivers with respite, to ensure that caregivers did not use all their paid time off, and when there were unavoidable scheduling conflicts. Interestingly, several caregivers still discussed not wanting to burden anyone by asking for help. In many cases caregivers described instances when others had asked specifically if they could help by providing transportation.

Not all caregivers experienced the support they required, as some described desiring help from family members or friends but sometimes patients did not want the caregivers to ask for help or burden others.

#### 3.2.3 Institutional and/or civic solutions to transportation support (*n* = 84; 58%)

Caregivers discussed requiring assistance with transportation and rather than looking to individual solutions many described the benefits that a patient transportation service or receipt of care at community satellite clinics would provide.

Others did not have regular access to personal transportation and had to resort to using an ambulance, taxi, or public transportation. Caregivers discussed having unreliable access to shared vehicles and vehicles in poor condition that often necessitated the use of public transportation. However public transportation was not always the best solution due to costs, patient discomfort and caregiver concern over patient safety. These issues were particularly problematic as the patient's condition worsened and the caregiver's ability to maneuver the patient into and out of vehicles became more difficult. Caregivers discussed the need for reliable modes of transportation designed for patients so that they were comfortable, included assistance getting in and out and could be coordinated easily and reliably.

#### 3.2.4 Expressions of care through acts of service (*n* = 68; 47.5%)

Being able to regularly provide transportation to medical appointments and treatments was also viewed as a source of pride and feeling that they have made sure that the patient has received the best care. This was especially true given the great number of scheduling and time barriers caregivers discussed having to overcome.

Seeing patients feel better because of the help of the caregiver was also rewarding, for example, caregivers discussed driving patients to hair appointments, to visit friends and to complete other errands they wished to do on their own but could no longer drive.

## 4 DISCUSSION

Transportation costs pose a significant challenge to caregivers. We identified an average transportation cost of $43.6 in each 2‐week period based on receipts submitted by the caregiver participants, an amount that could accumulate substantially depending on the duration of transportation costs occurring. For example, a caregiver providing transportation for 3 months could be expected to expend about $130 and for 6 months about $260. And of course, that is in addition to other expenses associated with cancer caregiving. In addition to the monetary or direct financial challenges transportation posed, caregivers also discussed opportunity costs which we defined as indirect financial costs. It is important to note that these are caregiver‐ and not patient‐identified challenges, which may help to explain this nuance. Studies describing the negative effects of transportation distance or costs on cancer care receipt and health outcomes are typically from the perspective of the patient.^5^ For caregivers, opportunity costs are often overlooked but may have important implications for caregiver's economic health. For example, managing transportation schedules and having to spend several hours at the cancer center detracted from time spent at work and seeking opportunities for growth in employment. Results from the mixed method analysis suggest new, more nuanced challenges faced by cancer caregivers specifically pertaining to transportation, an often cited barrier to seeking and receipt of optimal cancer care.^1^, ^5^ Caregivers also provide suggestions for institutional and/or civic based solutions rather than individual‐level intervention.

This work highlights the difference between costs as a burden versus a barrier to care, a distinction that requires greater definition in the literature. Patients and caregivers with greater resources likely are insulated from transportation costs acting as a barrier to obtaining care yet we found that they still recognize these OOP costs as burdensome. Additionally, this work highlights that the perceived burden of less commonly considered costs to the family of treatment, transportation costs was present regardless of family resources. Thus, perceived burden and financial hardship from transportation costs are distinct. Importantly, it was the time spent traveling caregivers mentioned as problematic, meaning that the time or opportunity costs were high, in addition to the actual outlays on transportation. These results provide additional context to a growing but unclear literature about transportation as a barrier to quality cancer care. While some work has found greater distance from the cancer center negatively impacts access to care such as receipt of timely cancer screenings, completion of cancer treatments and clinical trial participation.^1^, ^5^, ^21^ Other studies have not seen these relationships.^22^ One qualitative study found that while caregivers wanted patients to receive the best care possible burdens associated with clinical trial participation remain unaddressed, including traveling and scheduling appoints, suggesting that caregiver perceptions of burden are important factors in care planning and decision making.^23^ In a study of community health navigators transportation was identified as a topic for which they provide assistance but was not a priority for the majority of clients.^24^ Taken together these results suggest that the definition (barrier or burden) and context (patient or caregiver) for which transportation is viewed is critical.

Current advances in transportation are heavily geared towards identified solutions for efficiency and economic development, including expansion of road systems, driver safety, and emission reduction.^3^ While caregivers in this study did not utilize the services offered the Road to Recovery program (potentially influenced by the COVID‐19 pandemic), the program outlines a possible solution for barriers to transportation access via collaboration between public and private sectors. Increasing the numbers of individual drivers through rideshare technology and volunteers can alleviate some burden from caregivers and improve health outcomes for patients. A critical caveat is that this solution does not address accessibility for patients, a point raised by many cancer caregivers. It is unknown whether barriers in addition to the pandemic affected access and use but further examination is needed to inform how such programs could be successfully implemented. However, given the extent to which transportation is identified as an obstacle it may be that improvements require systems‐level approaches that leverage innovative collaborations between cancer centers, policy makers, urban planners, and physicians. Future interventions may include partnerships that enable the provision of specially equipped busses that serve the cancer center/hospital route and focus on expanding public transit routes, lengthening service hours, and discounts on passes. On the healthcare end, cancer centers could increase flexibility with appointment hours and scheduling exceptions for example among those who live further away and may require travel time allowances, and through expansion of sponsored rideshare programs.

### 4.1 Limitations

While it is possible that caregivers may have forgotten or chosen to exclude some receipts leading to incomplete collection of OOP costs this was minimized through the regularity of the biweekly collection including a one‐on‐one discussion of receipts with a study team member at each visit. It is notable that this collection method allowed the inclusion of nonmedical costs which are critical in order to develop a more nuanced understanding of the total costs of cancer caregiving.

## 5 CONCLUSION

Transportation costs have limited access to appropriate health care among cancer patients and encumbered those tasked with providing transport. Among caregivers in this sample the perception of transportation posing financial burdens was associated with the monetary costs of transportation, even among more affluent participants. Indirect costs are by nature less tangible, such as time dedicated to transport rather than personal wellbeing, employment, and other individual duties. The definition of barrier versus burden and their respective role in the successful provision of cancer care require further examination, including. The most salient challenges associated with how transportation barriers and burdens are experienced also likely depends on the participant role (e.g., patient or caregiver). Despite the nuances in definition, transportation costs noticeably play a role in cancer care with caregivers requesting broader, institutional changes to decrease monetary and non‐monetary costs.

## AUTHOR CONTRIBUTIONS

**Maria D. Thomson:** Conceptualization (equal); formal analysis (lead); methodology (equal); supervision (equal); writing – original draft (lead); writing – review and editing (lead). **Courtney Harold Van Houtven:** Formal analysis (equal); funding acquisition (supporting); investigation (equal); methodology (equal); supervision (supporting); writing – review and editing (supporting). **Rebecca Xu:** Writing – original draft (supporting); writing – review and editing (equal). **Laura A Siminoff:** Conceptualization (lead); formal analysis (supporting); funding acquisition (lead); investigation (equal); methodology (lead); writing – original draft (supporting); writing – review and editing (supporting).

## FUNDING INFORMATION

NCI (R01CA196576).

## CONFLICT OF INTEREST STATEMENT

The authors made no disclosures.

## Supporting information


Data S1:

## Data Availability

The data that supports the findings of this study are available from the corresponding author, MDT, upon reasonable request.
