# Supplementary material for: The many “costs” of transportation: Examining what cancer caregivers experience as transportation obstacles
Source: Cancer Med. 2023 Jul 23;12(16):17356–64. doi: 10.1002/cam4.6351 (PMC10501274; doi:10.1002/cam4.6351)
Supplement: Supplementary file 1 — Data S1: [file CAM4-12-17356-s001.docx]

**Part 1**

1. Can you tell me a little bit about how things have been since I last saw you? How have you been doing?

1. In the last two weeks, has anything changed about your living situation?

1. In the last two weeks, what has been most rewarding about caring for [PATIENT NAME]? What do you think you do well?

1. In the last two weeks, what caregiving tasks and responsibilities have you found to be the most difficult? **[PROBE: What makes you uncomfortable, scares or worries you?]**

1. If money was no object, or the service was free, what would you like a professional to do?  [**PROBE: Why those particular tasks and not others?]**

1. How would you describe the support you have been getting from others in the last two weeks? What are some examples of support that you have or haven’t received? Are you satisfied with the support you’re getting from others? **[PROBE: Do you wish you were getting more or less support? What kind of support would you consider helpful?]**

**Part 2**

*I know we just went over some tasks you may have been doing. Now, I want to specifically ask about the activities that you are doing because of [PATIENT NAME]’s illness. These next questions are only about tasks you did in the past 24-hours and how long you spent doing them. Please answer each question to the best of your ability.*

*In the past 24-hours, did you…*

|  |  |  |  | **HOW LONG DID YOU SPEND?** | |
| --- | --- | --- | --- | --- | --- |
|  | No  (0) | Yes  (1) |  | Hours | Minutes |
|  |  |  | Prepare and/or clean up meals |  |  |
|  |  |  | Do housework |  |  |
|  |  |  | Do laundry |  |  |
|  |  |  | Work on yard or house maintenance |  |  |
|  |  |  | Shop for groceries or other necessities |  |  |
|  |  |  | Provide transportation |  |  |
|  |  |  | Go with [PATIENT NAME] to an appointment |  |  |
|  |  |  | Pay medical bills |  |  |
|  |  |  | Spend time on the telephone with insurance company |  |  |
|  |  |  | Arrange appointments, medical care, visitors, or supplies, etc. |  |  |
|  |  |  | Spend time on the phone with billing department (hospital, doctor’s office) |  |  |
|  |  |  | Help [PATIENT NAME] with dressing or bathing |  |  |
|  |  |  | Help [PATIENT NAME] with walking |  |  |
|  |  |  | Help [PATIENT NAME] with toileting/incontinence/diaper care |  |  |
|  |  |  | Help [PATIENT NAME] with moving in or from bed or chair |  |  |
|  |  |  | Help [PATIENT NAME] with problem symptoms or side effects |  |  |
|  |  |  | Take care of a feeding machine, catheter, colostomy |  |  |
|  |  |  | Hook up or unhooked an IV |  |  |
|  |  |  | Give oral medications (**except** pain medication) |  |  |
|  |  |  | Give medicine by injection |  |  |
|  |  |  | Give pain medication |  |  |
|  |  |  | Take care of wounds or bed sores |  |  |
|  |  |  | Sit with [PATIENT NAME] alone (watching TV, reading, talking) |  |  |
|  |  |  | Sit with [PATIENT NAME] and other family/friends |  |  |
|  |  |  | Any other caregiving tasks or activities (please describe) |  |  |
